# Reliability of subjective evaluation in assessing elite table tennis players’ performance

**DOI:** 10.3389/fpsyg.2025.1432711

**Published:** 2025-06-30

**Authors:** Lulu Gan, Jing Chen, Luning Wang, Yunfei Lu, Jie Ren

**Affiliations:** ^1^China Table Tennis College, Shanghai University of Sport, Shanghai, China; ^2^Division of Health Professions, Texas A&M University–Texarkana, Texarkana, TX, United States; ^3^School of Psychology, Shanghai University of Sport, Shanghai, China

**Keywords:** sports performance, table tennis, elite athlete, reliability, subjective evaluation

## Abstract

**Purpose:**

This study aims to assess the reliability of subjective evaluations conducted under two information conditions and to explore the influence of observer expertise on the consistency of performance assessments of elite table tennis players.

**Methods:**

Observers of varying skill levels were invited to provide subjective evaluations of the elite table tennis players’ performance by observing specific rally strokes during the match. A Video Masking Paradigm approach was implemented to conceal motion information during critical moments of scoring and losing. The weighted Kappa coefficient (*k*) was employed to evaluate the inter-observer consistency between two observers. The Kendall’s coefficient of concordance (*w*) is a measure of inter-rater agreement, specifically used for ordinal scales (e.g., Likert five-point scale) when multiple raters are involved.

**Results:**

Intra-observer reliability was good (*r* = 0.61–0.86), whereas inter-observer consistency between the two observers was low (*k* = 0.01–0.39). Among the observation indicators, the advanced group showed the lowest consistency in evaluating tactical behavior (without results, *w* = 0.44; with results, *w* = 0.76). Experiment 2: The consistency of the observers in the without results condition (expert group *w* = 0.75 vs. advanced group *w* = 0.57 vs. novice group *w* = 0.66) is lower than in the with results (expert group *w* = 0.84 vs. advanced group *w* = 0.78 vs. novice group *w* = 0.76). Across all three observation indicators, namely stroke quality, tactical intention, and competitive posture, the expert group demonstrated the highest level of consistency, followed by the advanced group, while the novice group exhibited the lowest level of agreement.

**Conclusion:**

Observers with table tennis skill levels demonstrate high intra-observer test–retest reliability in subjective evaluations, but the inter-observer consistency is lower. Different information conditions (with or without results) are key variables affecting the consistency of subjective evaluations. When kinematic information is occluded (without results), the consistency of subjective evaluations decreases. The selection of observation indicators also impacts the consistency of subjective evaluations. Additionally, observers’ consistency in subjective evaluations is influenced by their level of experience and skill: the higher the observer’s level and experience, the greater the consistency of their subjective evaluations.

## 1 Introduction

Sports performance analysis (SPA) involves a systematic process in which researchers employ observational methods to document and assess athletes’ performance during competitions or training sessions (O'Donoghue, 2010). Originally known as notational analysis (Hughes and Franks, 1997), early SPA relied on symbols and other indicators to record observable behaviors and events in real-time scenarios, such as tallying the number of successful or unsuccessful basketball shots. Over time, motion analysis has become increasingly popular. This method primarily utilizes technologies such as photography, video, or wearable devices to collect and quantitatively analyze kinematic indicators such as athletes’ limb movements, including running distance (Kilit and Arslan, 2017), speed (Ibrahim et al., 2022), and limb movements (Jin and Ren, 2025).

Within table tennis, the SPA predominantly focuses on technical and tactical game analysis, often involving the statistical evaluation of points won and lost, as well as the number of strokes, particularly during the closing phases of matches. Notable methods include the three-segment indicator assessment (Wu, 2004) and its derivatives, such as the four-segment indicator assessment method (Yang and Zhang, 2014), the dual-system five-segment assessment (Jiang and Yao, 2015), and other dynamic statistical approaches (Zhang et al., 2018; Xiao et al., 2018). However, some scholars argue that relying solely on success rates (scoring and losing) to evaluate the technical efficacy of each stroke or rally may fail to capture the complexities of a table tennis match (Yi and Li, 2023).

The integration of advanced technologies, such as high-precision sensors, wearable devices, motion recognition, artificial intelligence, and big data analytics, has greatly enhanced SPA in table tennis. For example, researchers utilize high-speed video cameras (Iino and Kojima, 2011), infrared capture (Malagoli Lanzoni et al., 2018), force tables (Lam et al., 2019), and specialized equipment like table tennis eagle-eye systems to measure and analyze athletes’ swing speeds (Wu et al., 2021), movement patterns, and stroke quality. However, research using these technologies is typically conducted in a controlled laboratory environment, which may not accurately replicate the dynamic competitive scenarios in the real world, especially in table tennis, where each shot the athlete hits involves tactical intentions of control and counter-control (Wu, 2004).

Given the multifaceted nature of tactical and technical performance in table tennis, relying solely on notational and movement analysis may not reveal some of the sport’s unique patterns. Therefore, subjective evaluation methods serve as crucial complementary tools (Krizkova et al., 2021). Combining quantitative and qualitative analyses is essential for a comprehensive understanding of table tennis players’ performance (Wu et al., 2017). Therefore, subjective evaluation methods, such as expert observation of stroke quality or coaches’ real-time feedback during training and competitions (such as real-time off-field guidance on athletes’ performance during time-outs and gaps during the match), play a vital role in SPA (Zhang, 2004). In competitive sports such as tennis, badminton, pickleball, and other ball games, on-court guidance primarily relies on coaches’ direct observation and subjective evaluation. In contrast, disciplines like gymnastics, diving, and Wushu determine performance outcomes through judges’ subjective scoring. Moreover, feedback—whether objective (e.g., knowledge of results) or subjective (e.g., perceived quality of performance)—can originate from either internal sources (e.g., the athlete) or external sources (e.g., coaches, teammates, or officials), depending on the specific characteristics of each sport.

Various studies have been conducted to subjectively evaluate aspects of table tennis strokes, including their type (Lanzoni et al., 2014), effects (Wang, 2021), and indicators of individual techniques and combinations (Grycan et al., 2022), with observers providing assessments. These evaluations are then validated for reliability using statistical methods. While the reliability of these studies is generally high, simple evaluations and classifications by observers may not fully reveal the subtle differences in an athlete’s competitive abilities during a match. Moreover, solely depending on match results to assess an athlete’s performance cannot fully reflect their true competitive state. For example, observers may judge based only on actions or shot outcomes, but such simplified evaluations often overlook deeper technical and tactical strategies. Scores and points are merely surface-level results of each rally, while important factors such as tactical behavior, stroke quality, and how athletes achieve an advantageous competitive posture in the match are often not adequately reflected by the final shot outcome. Therefore, greater emphasis should be placed on the subjective evaluation of players’ performance to understand their abilities and skills comprehensively.

Based on the inherent characteristics of table tennis and the trends in developing its tactics and techniques, macro indicators can be divided into two categories: the serving system and the receiving system (Jiang and Yao, 2015). This study selects key stroke sequences from the serving system (strokes 3 and 5) and the receiving system (strokes 2 and 4) for subjective evaluation. Strokes 2 and 3 represent the athlete’s ability to initiate aggressive play from the receive and post-serve phases, respectively, with the ability to attack and gain advantageous competitive positions being crucial proactively. Strokes 4 and 5, on the other hand, represent the transition ability following the aggressive play from both receiving and serving, and these strokes are key points in the transition between attack and defense in a table tennis match.

This study aims to investigate the reliability of subjective evaluations in assessing performance indicators that are inherently difficult to quantify, such as stroke quality, tactical behavior, and competitive posture. Specifically, it examines whether the consistency of these evaluations is affected by the occlusion of shot outcomes. In addition, the study explores whether observers’ level of expertise influences the consistency of their subjective judgments.

## 2 Methods

### 2.1 Participants

Recruitment was conducted through announcements and direct invitations within the university’s table tennis team. Inclusion criteria were based on the 2021 “Table Tennis Athlete Technical Level Standards” issued by the General Administration of Sport of China (2021), which served to classify observers according to their skill level. All participants were from the same sports university and had a basic theoretical understanding of table tennis techniques and tactics. Detailed grouping information is presented in Table 1.

**Table 1:** Basic information of participants.

Experiment	Level	Group	Total *n*	Experience
1	First Level	Advanced Observers	6	3 years
2	National	Expert Observers	4	5 years
	First Level	Advanced Observers	4	3 years
	Second Level	Novice	4	0 years

The study is divided into two experiments. In Experiment 1, six first-level table tennis athletes were selected as advanced observers (3 males and 3 females, aged 22.83 ± 1.21 years), all of whom had 3 years of experience in tactical and technical analysis. In Experiment 2, to further verify the reliability of observers with different skill levels, we recruited 4 national-level observers (2 males and 2 females, aged 23.75 ± 0.61 years), all with five or more years of experience in tactical and technical analysis, referred to as expert observers, and 4 s-level observers (2 males and 2 females, aged 20.15 ± 0.95 years), who had no formal tactical or technical analysis experience, referred to as novice observers. At the same time, to compare the reliability across groups, we randomly selected the evaluation data of four first-level observers from Experiment 1. These observers included two males (Observers 04 and 05) and two females (Observers 02 and 03). Random selection was performed using the RAND() function in Microsoft Excel. Each eligible first-level observer from Experiment 1 was assigned a random value using = RAND(), and the observers were then sorted based on these values. To ensure gender balance consistent with the expert and novice observer groups, the top two males and top two females in the sorted list were selected for inclusion in the comparative analysis.

### 2.2 Test materials data resources

Observers watched edited video clips featuring four singles matches from the 2022 World Table Tennis (WTT) World Cup Finals between Sun Yingsha (China) and Tomokazu Harimoto (Japan), as outlined in Table 2. The edited videos maintained the camera’s angle, distance, and resolution (1920 × 1,080 pixels), and were free from advertisements. Since these were recorded videos, observers rated the matches from a side-view perspective (from the umpire’s position).

**Table 2:** Types of video materials.

Number	Name of the competition	Battle	Score
1	2022 WTT World Cup Men’s Singles Final	Zhang vs. Wang Chuqin (China)	2:4
2	2022 WTT World Cup Men’s Singles Semi-Finals	Zhang vs. Ochanov (Germany)	4:0
3	2022 WTT World Cup Women’s Singles Final	Sun vs. Chen Meng (China)	4:3
4	2022 WTT World Cup Women’s Singles Semi-Finals	Sun vs. Wang Yidi (China)	4:1

Each video clip encapsulated a single point (round) of play. The video length for each clip began 2 s before the serve and ended when the rally was dead—that is, when the athlete completed the final stroke and the outcome of the point was determined (e.g., the opponent failed to return the shot, the ball bounced twice, or the point was otherwise concluded). The video materials were divided into two categories: Class A (scoring information/match results known) and Class B (scoring information/match results unknown). Class A included 48 video clips (*n* = 48). Each evaluator reviewed 24 videos (*n* = 24) per player, including successful scores (*n* = 12; the ball landed on the opponent’s side and resulted in a point) and losing scores (*n* = 12; the ball was not successfully returned or did not land on the opponent’s side). Each video contained four battle rounds (*n* = 4). Subsequently, a set of Class B videos was edited from the Class A footage by removing the final strokes after the rallies. These Class B videos (*n* = 48) were further edited using a Time Video Masking Paradigm (VMP) to obscure key kinematic information (e.g., masking the final shot result of each rally) (Farrow et al., 2005), making it difficult to determine the winner.

For instance, to evaluate the characteristics of Sun Yingsha’s fourth shot, the Class A scoring clip would depict the opponent losing the point on the fifth shot, while the Class A losing clip would show Sun Yingsha losing the point on the sixth shot, representing an inverse scenario of the fourth shot characteristics. Conversely, the Class B video would solely display Sun Yingsha hitting the fourth stroke ball onto the table, without revealing whether the opponent’s return ball landed on the table. The video material was edited and categorized into two types, as outlined in Table 3, with “effective hit” referring to the ball successfully landing on the opponent’s side of the table.

**Table 3:** Experimental video types.

Athletes	Evaluation round	Stroke results	Video masking position		Number of videos
			A	B	
Zhang	3	Scoring	An effective hit on the 3rd stroke, followed by your opponent losing the point on the 4th stroke.	An effective hit on the 3rd stroke, with the ball bouncing on the second bounce and the opponent failing to touch it.	3
		Losing	The opponent’s effective hit on the 4th stroke, after Zhang loses the point on the 5th stroke.		3
	4	Scoring	An effective hit on the 4th stroke, followed by your opponent losing the point on the 5th stroke.	An effective hit on the 4th stroke, with the ball bouncing on the second bounce and the opponent failing to touch it.	3
		Losing	The opponent’s effective hit on the 5th stroke, after Zhang loses the point on the 6th stroke.		3

All the videos were sourced from the replay recordings on the China Migu Video App and were screened by two national-level table tennis athletes and researchers to prevent observer judgments from being influenced by other factors, such as scoring or faults (e.g., clear out-of-bounds, net touches, or failure to make contact with the ball).

### 2.3 Definitions of observation indicators

#### 2.3.1 Stroke quality

Stroke quality consists of five key physical elements: speed, power, spin, placement, and trajectory. When evaluating stroke quality, observers should assess all five elements as a whole, rather than focusing on a single factor in isolation. This is because the quantified combination of individual elements can result in up to 14 million variations of ball characteristics, underscoring the importance of a holistic evaluation (Wu, 2004). A high-quality stroke is characterized by fast execution, significant power, strong spin, low trajectory, and precise placement (Wu, 2004), whereas the opposite indicates low stroke quality.

#### 2.3.2 Tactical behavior

Tactical behavior, representing the dynamic interplay of athletes with a plethora of techniques and tactics, is critical (Yue, 2017). The first serve in a match represents a critical tactical behavior. An effective serve can prevent an opponent’s direct attack, thereby allowing the server to transition to an offensive position by the third stroke. It reflects a clear demonstration of both control and counter-control intentions. For example, skilled athletes exhibit a strong offensive mindset during the serve phase, a clear intent to restrict the opponent’s tactical execution during the receive phase, and noticeable variations in rhythm or trajectory throughout the progression of the match (Zhang, 2004).

#### 2.3.3 Competitive posture

Competitive posture, comprising active, passive, and stalemate forms on the field (Zhang, 2004), can be categorized into five levels based on performance: Excellent, Good, Moderate, Poor, and Very Poor. An excellent stroke creates a significant scoring opportunity or gives the player a clear advantage in terms of control. A good stroke provides the player with a certain degree of initiative, establishing a solid foundation for subsequent offensive or defensive actions. A neutral stroke maintains an equilibrium in the contest, offering no clear advantage to either player. A poor stroke may allow the opponent to gain the initiative, placing the player in a passive position. A very poor stroke can directly result in the opponent obtaining a significant scoring opportunity or a clear advantage.

### 2.4 Experimental design

To minimize potential observer bias, all observers were blinded to the specific objectives and hypotheses of the study. Interactions between researchers and observers were standardized and restricted solely to clarifying operational definitions and experimental procedures. No feedback or guidance was provided concerning scoring criteria or expected outcomes. Before the formal experiment, observers engaged in simulated scoring sessions to ensure a comprehensive understanding of the operational procedures. They were also required to review the full experimental protocol and sign a confirmation form affirming their comprehension of the observation indicators and task requirements.

Throughout the experiment, observers followed on-screen prompts to evaluate three key aspects of the target athlete (Sun or Zhang) during a designated rally: stroke quality, tactical behavior, and competitive posture. After viewing each video, they responded to the following questions: (1) What is your evaluation of Sun or Zhang’s Xth stroke in this rally? (2) How would you assess the athlete’s tactical behavior? (3) How would you assess the athlete’s competitive posture? Observers provided subjective ratings using a five-point Likert scale (5 = Excellent, 4 = Good, 3 = Fair, 2 = Poor, 1 = Very Poor), based on their individual perceptions of performance in each dimension.

During the formal experimental phase, all observers were allowed to independently watch the videos multiple times and to evaluate each technical and tactical analysis indicator subjectively. Observers were not subject to any standardized limits on the number of rewatches or the duration of observation. The formal experiment is divided into two phases: The first phase consists of two parts. In the first part, observers undergo 8 simulation rating exercises and then evaluate 48 B-class videos, followed by a 5-min break. In the second part, observers also complete 8 simulation rating exercises and then evaluate 48 A-class videos. The simulation exercises were designed to help observers become familiar with the evaluation criteria and video format prior to formal assessment. Observers rated the sample videos using the same Likert scale and procedure applied in the formal experiment. Upon completion of the experiment, observers were required to complete a questionnaire assessing their adherence to the operational guidelines during the assessment process.

In the second phase, which occurs 3 weeks later, observers re-evaluate 24 randomly selected A-class videos and 24 B-class videos to assess the internal consistency among the observers. Random selection was performed using the RAND() function in Microsoft Excel. Each video clip in the Class A and Class B datasets was assigned a random value using = RAND(), and the clips were sorted accordingly. Random sampling was then conducted based on these values, while preserving the distributional integrity of the video dataset.

This two-phase design was applied in both Experiment 1 and Experiment 2, ensuring that subjective evaluations were examined across different information conditions and observer groups.

### 2.5 Statistics analysis

In Experiment 1, Pearson’s correlation coefficient (*r*-value) was used to assess the internal stability (test–retest reliability) of observers between two assessments. It is considered an indication of high internal reliability when the r-value exceeds 0.60 (Schober et al., 2018). The weighted Kappa coefficient (*k*-value) was used to evaluate inter-observer agreement between two observers. It is interpreted as high agreement when the k-value exceeds 0.60, moderate agreement when it falls between 0.40 and 0.60, and low agreement when below 0.40 (McDowell, 2006). The weighted Kappa coefficient is specifically designed to assess agreement on ordinal categorical data, as it accounts for differences between rating levels. It assigns greater weight to similar ratings and lower weight to larger discrepancies, making it particularly suitable for measuring observer rating consistency. Kendall’s coefficient of concordance (*W*) was employed to measure the consistency of observers in the advanced group under different information conditions (with or without outcome results) (Daniel, 1980; Marascuilo and McSweeney, 1977). The *W*-value ranges from 0 to 1, with values closer to 1 indicating higher consistency.

In Experiment 2, Kendall’s W was used to evaluate the consistency of observers at different expertise levels (expert group, advanced group, and novice group) under both information conditions, as well as their consistency across the three performance indicators.

## 3 Results

### 3.1 Retest reliability

The reliability of the observers’ assessments between the two tests is shown in Table 4. The correlation coefficients (*r* = 0.61–0.86) for the advanced observers’ subjective evaluations of stroke quality, tactical behavior, competitive posture, and overall assessment indicate a high level of internal reliability.

**Table 4:** Reliability test of the results of two ratings of the same video by advanced observers (*r*-value).

Observers	Stroke quality	Tactical behavior	Competitive posture	Overall evaluation
1	0.63	0.62	0.61	0.62
2	0.65	0.63	0.64	0.64
3	0.76	0.69	0.60	0.69
4	0.67	0.67	0.62	0.65
5	0.79	0.65	0.68	0.70
6	0.83	0.86	0.70	0.79

### 3.2 Consistency of inter-observer evaluations

The consistency between the two advanced observers is shown in Table 5. The kappa values for subjective evaluations of stroke quality, tactical behavior, competitive posture, and overall assessment (*k* = 0.01–0.39) indicate very low consistency between the two observers, suggesting low reliability in the subjective evaluations.

**Table 5:** Statistical results of the consistency test for advanced observers’ evaluation of Class B videos.

Observation indicators	Observers	*k*-value					
		1	2	3	4	5	6
Stroke quality	1	—					
	2	0.19	—				
	3	0.25	0.35	—			
	4	0.12	0.10	0.04	—		
	5	0.17	0.12	0.09	0.04	—	
	6	0.12	0.39	0.25	0.27	0.14	—
Tactical behavior	1	—					
	2	0.08	—				
	3	0.16	0.15	—			
	4	0.13	0.20	0.13	—		
	5	0.04	0.16	0.02	0.10	—	
	6	0.11	0.27	0.16	0.13	0.39	—
Competitive posture	1	—					
	2	0.26	—				
	3	0.13	0.21	—			
	4	0.15	0.30	0.15	—		
	5	0.12	0.01	0.06	0.01	—	
	6	0.15	0.27	0.30	0.13	0.14	—
Overall evaluation	1	—					
	2	0.20	—				
	3	0.18	0.23	—			
	4	0.16	0.23	0.11	—		
	5	0.11	0.09	0.03	0.05	—	
	6	0.13	0.31	0.23	0.18	0.11	—

### 3.3 Observation indicators consistency of subjective evaluation

**Figure 1:**
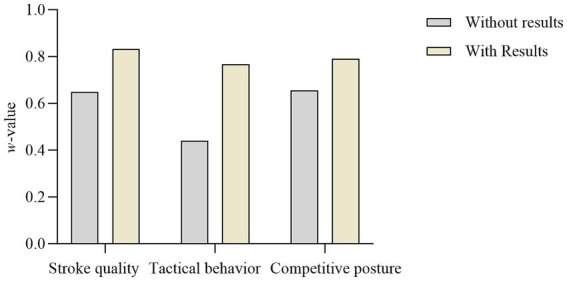
Statistical results of Kendall’s W for evaluations conducted by observers in Class A and Class B videos (*w*-value).

The consistency of advanced group observers’ evaluations for the three observation indicators under different kinematic information conditions is shown in Figure 1. In the without results condition, the consistency for stroke quality (*w* = 0.64) and competitive posture (*w* = 0.65) is higher than for tactical behavior (*w* = 0.44). In the with results condition, stroke quality (*w* = 0.83) shows the highest consistency, while tactical behavior (*w* = 0.76) shows the lowest consistency. This suggests that different observation indicators can influence the consistency of observers’ evaluations.

### 3.4 Consistency of subjective evaluations with and without stroke results

**Figure 2:**
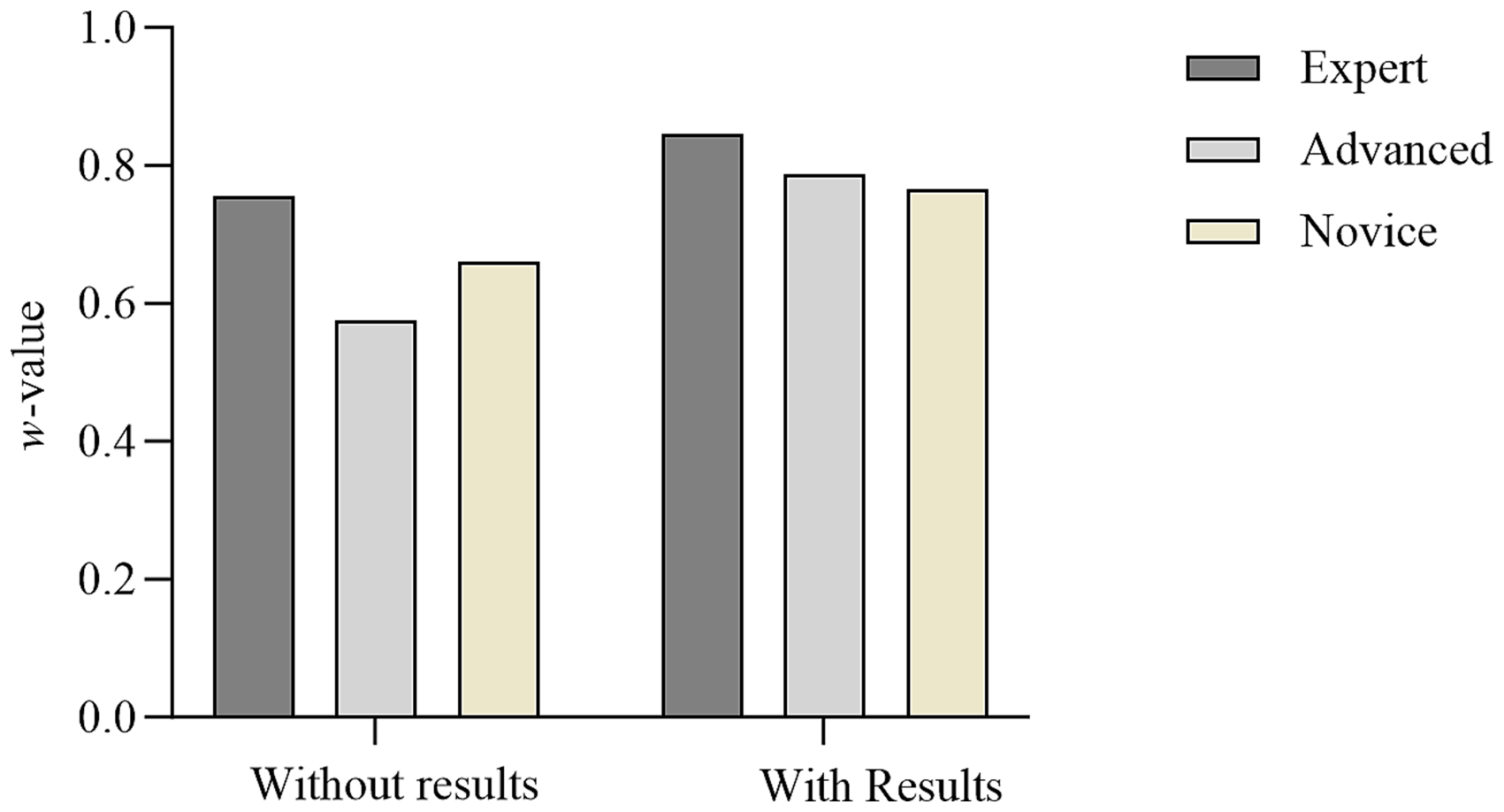
Statistical results of Kendall’s W for evaluations conducted by observers at different expertise levels in Class A and Class B videos (*w*-value).

The consistency of observers at different skill levels is shown in Figure 2. The consistency of the observers in the without results condition (expert group *w* = 0.75 vs. advanced group *w* = 0.57 vs. novice group *w* = 0.66) is lower than in the results (expert group *w* = 0.84 vs. advanced group *w* = 0.78 vs. novice group *w* = 0.76). This suggests that the subjective evaluations made by observers at different skill levels are influenced by occluded kinematic information related to the shot outcome.

### 3.5 Differences in consistency among observers of different levels

**Figure 3:**
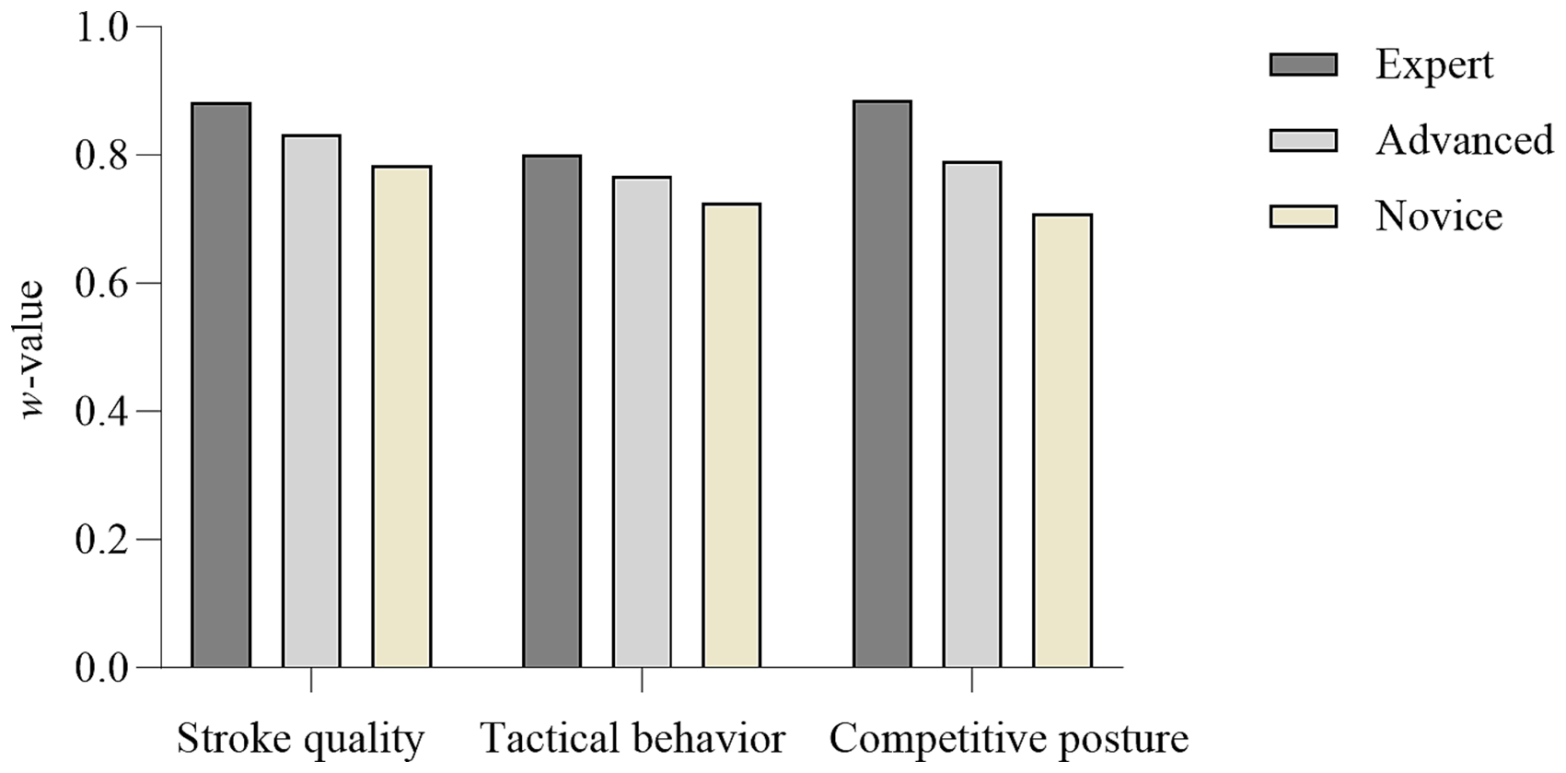
Statistical results of Kendall’s W for observation indicators rated by observers at different levels in Class B videos (*w*-value).

The consistency of observers at different skill levels when evaluating the three observation indicators is shown in Figure 3. For stroke quality ratings, the expert group (*w* = 0.88) showed the highest consistency, followed by the advanced group (*w* = 0.83) and the novice group (*w* = 0.78). For tactical behavior ratings, the expert group (*w* = 0.80) had the highest consistency, followed by the advanced group (*w* = 0.76) and the novice group (*w* = 0.72). For competitive posture ratings, the expert group (*w* = 0.89) exhibited the highest consistency, followed by the advanced group (*w* = 0.79) and the novice group (*w* = 0.70). Among the three observation indicators, the expert group demonstrated the highest consistency, the first-level group showed moderate consistency, and the second-level group exhibited the lowest consistency. This suggests that the skill level of the observer affects the consistency of evaluations.

## 4 Discussion

### 4.1 Reliability of subjective evaluations

This study investigated whether observers’ evaluations could reach consensus using a five-level rating system (e.g., good, moderate, poor). While subjective evaluations are commonly employed in table tennis coaching, their reliability remains debated. Some argue that subjective evaluation methods lack statistical significance in analyzing techniques (Wu et al., 2017), while others report high intra-observer reliability in assessing hitting effectiveness (Wang, 2021). Our findings reveal high intra-observer reliability but low inter-observer consistency, suggesting that subjective evaluations may have limited reliability. Although the observers in this study were experienced, their lack of long-term relationships with the athletes may have influenced their ability to make precise evaluations (Davis et al., 2018). Additionally, the side-view recordings used may have differed from the typical direct or rear views, potentially affecting evaluation accuracy. The absence of contextual factors that are present in live competitions further diminishes the reliability of subjective assessments (Grycan et al., 2022). Moreover, differences in observers’ coaching styles and tactical strategies likely impacted their evaluations (O'Neil and Hodge, 2020; Salazar et al., 2024). These findings suggest that caution is warranted when using subjective evaluation methods in research.

### 4.2 Effect of stroke results on the consistency of subjective evaluation

Current research in table tennis typically measures player performance based on the score of the final shot in each round. However, this approach may be overly simplistic. Therefore, this study presents observers with specific performance phases of skilled athletes through the VMP. The objective is to investigate whether observers’ subjective evaluations are influenced by stroke results (with and without). Observers show higher consistency when they are aware of the stroke results, and research has shown that the use of VMP, along with explicit contextual priors, enhances the reliability of evaluations (Gredin et al., 2021). In prior studies on subjective evaluations in table tennis (Wang, 2021), the high consistency observed may be attributed to the reliance on stroke results, with observers inferring stroke quality from those results. Specifically, when a point is scored, observers tend to give a positive evaluation, whereas losing a point results in a negative evaluation. It is important to note that there is an inherent relationship between the previous and subsequent strokes (Yue, 2017), which cannot be directly equated to a singular performance result (i.e., scoring or losing a point). This study highlights the need for a more comprehensive evaluation that takes overall performance into account instead of just immediate match results, an aspect that has often been overlooked in prior research.

### 4.3 Effect of observational indicators on the consistency of subjective evaluations

Observers show high consistency in evaluating metrics like stroke type and direction (Lanzoni et al., 2014). However, when assessing tactical behavior, evaluations tend to diverge. From the perspective of the visual system’s judgment of technical movements, stroke quality, and competitive posture, indicators may be directly observable. For instance, stroke quality can be assessed by evaluating the arc height and placement of the ball. Additionally, a player’s choice to execute an offensive technique, such as forehand or backhand topspin, indicates a competitive posture with the initiative (Lanzoni et al., 2014). Tactical behavior involves complex thought, judgment, and decision-making (Zhang et al., 2018), and observers infer the underlying intentions from observable actions, often influenced by personal interpretation (Yang and Zhang, 2016).

### 4.4 Effect of observers’ levels on the consistency of subjective evaluations

This study found that observer experience and expertise are key factors in evaluation consistency. Experts demonstrated the highest consistency under both with and without result conditions. Experts rely on domain-specific knowledge to make stable and accurate evaluations (Ross et al., 2024), enabling them to more precisely judge the trajectory of the ball (Lu et al., 2020). In contrast, advanced observers exhibited lower consistency in the absence of result information, indicating that their evaluations are more reliant on external cues (Runswick et al., 2019). Interestingly, novice observers showed moderate consistency when result information was absent, outperforming advanced observers. This may be due to their reliance on simpler, more intuitive evaluation strategies, with less dependence on complex cues (Runswick et al., 2019).

### 4.5 Limitations

This study primarily focused on assessing the reliability of subjective evaluations rather than comparing performance across groups. Accordingly, the analysis centered on established reliability thresholds (e.g., Weighted Kappa, Kendall’s W) to evaluate intra- and inter-rater consistency. Descriptive statistics were reported to illustrate rating patterns, and no inferential statistical tests or *p*-values were provided, as group difference testing was beyond the scope of this study.

Moving forward, future research should build upon this reliability framework to conduct statistical comparisons across different groups or conditions. Furthermore, efforts to improve observers’ evaluative capabilities through targeted training and to refine and standardize evaluation criteria will be essential for enhancing the accuracy and validity of performance assessments. In addition, combining quantitative and qualitative approaches will provide a more comprehensive understanding of how athletes’ technical and tactical strategies affect competitive outcomes (Torres Luque et al., 2020).

## 5 Conclusion

Observers with table tennis skill levels demonstrate high intra-observer test–retest reliability in subjective evaluations, but the inter-observer consistency in subjective evaluations is lower, indicating that caution is needed when applying subjective evaluation methods. Different information conditions (with or without results) are key variables affecting the consistency of subjective evaluations. When kinematic information is occluded (without results), the consistency of subjective evaluations decreases. The selection of observation indicators also impacts the consistency of subjective evaluations. Observers’ consistency in subjective evaluations is influenced by their level of experience and skill; the higher the observer’s level and experience, the higher the consistency of their subjective evaluations.

## Data Availability

The raw data supporting the conclusions of this article will be made available by the authors, without undue reservation.
